# Musculo-skeletal phenotype of Costello syndrome and cardio-facio-cutaneous syndrome: insights on the functional assessment status

**DOI:** 10.1186/s13023-021-01674-y

**Published:** 2021-01-22

**Authors:** Chiara Leoni, Domenico Marco Romeo, Michele Pelliccioni, Mariangela Di Già, Roberta Onesimo, Valentina Giorgio, Elisabetta Flex, Marta Tedesco, Marco Tartaglia, Donato Rigante, Antonio Valassina, Giuseppe Zampino

**Affiliations:** 1 Center for Rare Diseases and Birth Defects, Department of Life Sciences and Public Health, Fondazione Policlinico Universitario A. Gemelli IRCCS, Largo Gemelli 8, 00168 Rome, Italy; 2 Pediatric Neurology Unit, Department of Life Sciences and Public Health, Fondazione Policlinico Universitario A. Gemelli IRCCS, Rome, Italy; 3 Department of Oncology and Molecular Medicine, Istituto Superiore Di Sanità, Rome, Italy; 4 Genetics and Rare Diseases Research Division, Ospedale Pediatrico Bambino Gesù, Rome, Italy; 5 Università Cattolica Sacro Cuore, Rome, Italy; 6 Unit of Neurophysiopathology and Sleep Medicine, Neurosciences and Orthopedics, Department of Geriatrics, Fondazione Policlinico Universitario A. Gemelli IRCCS, Rome, Italy

**Keywords:** Costello syndrome, Cardio-facio-cutaneous syndrome, Rasopathies, Musculo-skeletal profiling, Functional and disability assessment, Genotype–phenotype correlation, Patient-centered care, Innovative biotechnologies, Clinical biomarker, Personalized medicine, Tailored treatments

## Abstract

**Background:**

Costello syndrome (CS) and cardio-facio-cutaneous syndrome (CFCS) belong to the RASopathies, a group of neurodevelopmental disorders with skeletal anomalies. Due to their rarity, the characterization of the musculo-skeletal phenotype in both disorders has been poorly characterized.

**Patients and methods:**

Herein we reported data on orthopedic findings and functional status of a large sample of CS and CFCS patients. Thirty-four patients (CS = 17 and CFCS = 17) were recruited. Functional and disability evaluations were performed by assessing the 6-min walking test (6MWT) and Pediatric Outcomes Data Collection Instrument (PODCI). Genotype/phenotype correlation was also provided.

**Results:**

Orthopedic manifestations are highly prevalent in CS and CFCS and overlap in the two disorders. Overall, patients with CS harboring the recurrent *HRAS* Gly12Ser substitution show a more severe skeletal phenotype compared to patients carrying the Gly12Ala and Gly13Cys variants. Among CFCS patients, those with the *MAP2K1/2* variant show different skeletal characteristics compared to *BRAF* variants, with a higher prevalence of orthopedic abnormalities. Functional assessment showed that patients with CS and CFCS reached lower values compared to the general population, with CFCS patients displaying the lowest scores.

**Conclusions:**

Orthopedic manifestations appear universal features of CS and CFCS and they can evolve across patients’ life. Longitudinal assessment of disability status by using 6MWT and PODCI could be useful to evaluate the functional impact of orthopedic manifestations on patients’ outcome and help planning a tailored treatment of these comorbidities.

## Background

Costello syndrome (CS, OMIM #218040) and cardio-facio-cutaneous syndrome (CFCS, OMIM PS115150) are neurodevelopmental disorders caused by gain-of-function mutations in genes encoding components of the RAS/mitogen-activated protein kinase (MAPK) pathway, an intracellular signaling cascade playing a pivotal role in cell cycle regulation, differentiation, growth and senescence [1]. These conditions are grouped under the term RASopathies together with Noonan syndrome (NS) and neurofibromatosis type 1 (NF1) and other emerging clinically related disorders, due to a common pathogenetic mechanism resulting in dysregulation of the RAS/MAPK pathway [2]. Both CS and CFCS are ultra-rare conditions with approximately 300 individuals each reported worldwide [3, 4]. CS is caused by heterozygous activating mutations in the *HRAS* gene, with a missense change resulting in the Gly12Ser substitution, which represents the most common event underlying the disorder. As a consequence, CS is characterized by a relatively homogeneous phenotype [3]. Conversely, CFCS is genetically heterogeneous and caused by heterozygous activating mutations in the *BRAF, MAP2K1, MAP2K2* and *KRAS* genes, leading to a more variable clinical presentation [4]. Overall, key-features affecting RASopathies include a distinctive facial appearance, postnatal growth failure, a wide spectrum of cardiac defects, variable intellectual disability, skin manifestations and different signs of musculo-skeletal involvement. The musculo-skeletal profiling of NS and NF1 syndrome has previously been outlined in the medical literature [5–7], while incidence rates are lacking for both CS and CFCS, possibly because their overall rarity. Indeed, available data on skeletal anomalies in CS and CFCS have been collected during the International Costello Syndrome Conferences [8–14], but poor information is available about the function and disability level [8, 15] or daily living activities in these patients [16].

The present study aimed to improve our general knowledge about the musculo-skeletal phenotype of CS and CFCS thorough the assessment of a single, unselected and relatively large monocentric cohort of patients, and to add information about the functional status of such patients. Moreover, we discuss on the genotype/phenotype correlations found.

## Patients and methods

A prospective study was performed between May 2018 and January 2020. Patients with molecularly confirmed diagnosis of CS and CFCS managed in our Institution were consecutively recruited. A written informed consent was obtained from all participating individuals and families. The study was approved by the local Ethics Committee. All individuals underwent pediatric, genetic, neurological and orthopedic evaluations by physicians experienced in the management of RASopathies. Patients’ past medical history was reviewed in order to check for: (1) congenital orthopedic malformations; (2) achievement of neuro-motor developmental milestones; (3) use of orthosis; (4) physical activity; (5) congenital heart disease (CHD). Information about the cardiac status was collected to exclude CHD as a potential confounder for the poor results in functional assessments tests.

A standardized musculo-skeletal physical examination was carried out by filling in a comprehensive checklist, including 100 items (Additional file 1: Table S1). Coxa valga subluxans (CVS) was radiologically diagnosed when femoral neck-shaft angle was > 140°. Functional and disability evaluations were performed by assessing the 6-min walking test (6MWT) [17] in all patients with independent walking and by using the Pediatric Outcomes Data Collection Instrument (PODCI) [15, 18, 19]. The 6MWT was used to measure the distance covered on a flat and hard surface in a total of 6 min to monitor overtime worsening of general status and deambulation [17]. 6MWT data were normalized taking into account sex, age, body mass index and height [20, 21]. PODCI was used to assess the global (functional and psychological) profile of the orthopedic patient. Based on the global level of intellectual disability, PODCI was filled in by patients’ parents or caregivers; data on all PODCI domains (physical functions, transfer and basic mobility, sports, comfort, pain, happiness and satisfaction, global function) were collected.

Study data were analyzed with GraphPad PRISM software (version 5.1; GraphPad Software. Inc., San Diego CA, USA). Descriptive analysis was expressed as mean ± standard deviation (SD) and percentages. According to the cut-off age identified by PODCI, the whole sample was divided into two groups: children (when < 11 years), adolescents and young adults (if ≥ 11 years).

The nonparametric Mann Whitney test was used to compare orthopedic findings in CS and CFCS, PODCI scores between CS and CFCS and with normative values [19]*.* A *p* value < 0.05 was considered statistically significant.

## Results

A total of 34 patients with molecularly confirmed diagnosis of CS (N = 17) and CFCS (N = 17) were recruited in the study (CS: age range 4–36 years; CFCS: age range 5–35 years); most patients were older than 8 years (15/17 with CS and 12/17 with CFCS) (Table 1). No one patient reported in the present paper was ever enrolled in previous studies about orthopedic anomalies in CS and CFCS. Prevalence of congenital malformations detected at birth was very low (hip dysplasia 3/34, 9%; clubfoot 2/34, 6%) (Table 2). All patients experienced a delay in the neuromotor development milestones, which was treated with dedicated physical therapy, requiring the use of orthosis (mostly insoles, orthopedic shoes and ankle foot orthosis or AFO) in 15 (88%) CS patients and 13 (76%) CFCS patients (Table 2). At the time of clinical evaluation, all patients with CS reached head and trunk control, and 16/17 achieved an independent walking. In contrast, 6 (35%) patients with CFCS needed some kind of support for standing upright and/or start deambulation (orthosis and/or wheelchair for long distances). Most of patients played sport and/or performed physical therapy at the time of clinical evaluation (14/17, 82% CS; 13/17, 76% CFCS) (Table 2).

**Table 1:** Demographic and body mass index characteristics of the study population

Phenotype	Gene variant	No of patients	Age	BMI	Total
CS	*HRAS (p.Gly12Ser)*	14 (4 M; 10 F)	19.8 ± 9.7	19.2 ± 3.0	17 (6 M; 11 F)
	*HRAS (p.Gly13Cys)*	2 (1 M; 1 F)	14.5 ± 2.1	17.2 ± 0.2	
	*HRAS (p.Gly12Ala)*	1 (1 M; 0 F)	8	15.6	
CFCS	*BRAF (all variants)*^a^	13 (3 M; 10 F)	12.7 ± 6.2	16.7 ± 2.6	17 (6 M; 11 F)
	*MAP2K1(all variants)*^b^	3 (2 M; 1 F)	23.6 ± 9.0	15.0 ± 1.4	
	*MAP2K2(p.Ala62Pro)*	1 (1 M; 0 F)	7	17.4	

^a^BRAF variants: 3/13: p.Gln257Arg; 1/13: p.Val487Gly; 1/13: p.Thr599Ile; 1/13: p.Trp531Arg; 1/13: p.Thr470Pro; 1/13: p.Lys601Gln; 1/13: p.Leu525Pro; 1/13: p.Trp531Cys; 1/13: p.Lys483Asn; 1/13: p.Lys499Asn; 1/13: p.Gln709Arg

^b^MAP2K1 variants: 2/3: p.Tyr130Cys; 1/3: p.Leu42Phe

**Table 2:** Relevant medical findings

	CS N = 17 (%)	CFCS N = 17 (%)
*Congenital orthopedic malformation*		
Congenital hip dysplasia	2 (12)	1 (6)
Clubfoot	1 (6)	1 (6)
*Neuro-motor abilities*		
Head control	17 (100)	17 (100)
Trunk control	17 (100)	16 (94)
Autonomous deambulation^a^	17 (100)	11 (65)
Assisted Walking^a^	0 (0)	6 (35)
*Past use of orthosis*	15 (88)	13 (76)
*Use of orthosis*^a^		
DAFO/AFO	5 (29)	4 (24)
Postural Stroller	1 (6)	6 (35)
Corset/Belt/Brace	0 (0)	3 (18)
Orthopedic Insole	8 (47)	8 (47)
*Physical activity*^b^	14 (82)	13 (76)
Physical therapy	4 (24)	13 (76)
Sport	5 (29)	10 (59)
*Congenital heart disease*		
HCM	10 (59)	4 (23)
PVS	1 (6)	6 (35)
PVD	0 (0)	1 (6)
MVD	3 (18)	4 (23)
ASD/VSD	1 (6)	1 (6)

Prevalence data refers to acquired neuro-motor ability at time of clinical evaluation

*HCM* hypertrophic cardiomyopathy, *PVS* pulmonary valve stenosis, *PVD* pulmonary valve dysplasia, *MVD* mitral valve disease, *ASD* atrial septal defect, *VSD* ventricular septal defect

^a^Data showed at time of clinical evaluation

^b^We considered physical therapy or sport played ≥ 2 times per week

All patients showed a mild CHD which did not interfere with PODCI scales and 6MWT results. Only one patient with CS and a severe form of HCM underwent a surgical intervention of miectomy at 11 years. At the time of the present study, this patient was 19-year-old and she regularly performed sport three times per week without showing any clinical symptom.

The standardized checklist to detect musculo-skeletal manifestations revealed that most patients were affected by a variable combination of deformities. The most frequent osteo-articular abnormalities are shown in Table 3. The most frequent skeletal findings in CS were scoliosis, anterior chest anomalies, ulnar deviation of fingers, elbows contractures, cubitus valgus, feet anomalies (metatarsus varus, pes planus or cavus), Achille’s tight heel cord and small joint laxity. Age of onset of the syndromic scoliosis was after 10 years in 3/5 of patients (60%), and this was slowly progressive. Conversely, 2/5 of patients (40%) showed a moderate dystrophic scoliosis started at the age of 6 that did not require any surgical intervention. Both groups were regularly followed-up with yearly monitoring of overnight saturation and pulmonary function tests, which showed no significantly abnormal results during sleeping and a mild restrictive lung disease.

**Table 3:** Muscle-skeletal findings

	CS N = 17 (%)	*HRAS* Gly12Ser (N = 14)	*HRAS* Gly12Ala (N = 1)	*HRAS* Gly13Cys (N = 2)	CFCS N = 17 (%)	*BRAF* all variants (N = 13)	*MAP2K1/2* all variants (N = 4)
*Axial*							
Scoliosis	5 (29)	5 (36)	0 (0)	0 (0)	6 (35)	4 (31)	2 (50)
Pectus Carinatum/excavatum	8 (47)	8 (57)	0 (0)	0 (0)	9 (53)	6 (46)	3 (75)
Dorsal Hyperkyphosis	3 (18)	2 (14)	0 (0)	1 (50)	3 (18)	3 (23)	0 (0)
Stiff Back	3 (18)	2 (14)	0 (0)	1 (50)	3 (18)	3 (23)	0 (0)
Pterigium colli	0 (0)	0 (0)	0 (0)	0 (0)	6 (35)	5 (38)	1 (25)
Lumbar hyperlordosis	1 (6)	1 (7)	0 (0)	0 (0)	1 (6)	1 (8)	0 (0)
*Upper limbs*							
Fingers’ ulnar deviation	14 (82)	13 (93)	1 (100)	0 (0)	7 (41)	3 (23)	4 (100)
Elbows contractures	9 (53)	8 (57)	0 (0)	1 (50)	6 (35)	4 (31)	2 (50)
Cubitus valgus	6 (35)	4 (29)	0 (0)	2 (100)	4 (24)	3 (23)	1 (25)
Wrist contractures	2 (12)	2 (14)	0 (0)	0 (0)	1 (6)	0 (0)	1 (25)
Fingers’ radial deviation	0 (0)	0 (0)	0 (0)	0 (0)	1 (6)	1 (8)	0 (0)
*Lower limbs*							
Coxa valga subluxans^a^	8 (89)	7 (87)	1 (100)	–	3 (27)	3 (33)	0 (0)
Hip contractures	2 (12)	2 (14)	0 (0)	0 (0)	6 (35)	2 (15)	3 (75)
Adducted hip	2 (12)	1 (7)	0 (0)	1 (50)	3 (18)	1 (8)	2 (50)
Knees contractures	3 (18)	3 (21)	0 (0)	0 (0)	6 (35)	4 (31)	2 (50)
Genu valgum	5 (29)	5 (36)	0 (0)	0 (0)	3 (18)	1 (8)	2 (50)
Genu varum	0 (0)	0 (0)	0 (0)	0 (0)	2 (12)	1 (8)	1 (25)
Metatarsus varus	8 (47)	7 (50)	0 (0)	1 (50)	6 (35)	4 (31)	2 (50)
Pes valgus	4 (24)	4 (29)	0 (0)	0 (0)	9 (53)	6 (46)	3 (75)
Pes planus	6 (35)	5 (36)	1 (100)	0 (0)	6 (35)	4 (31)	2 (50)
Pes cavus	6 (35)	4 (29)	0 (0)	2 (100)	1 (6)	0 (0)	1 (25)
Hallux valgus	4 (24)	4 (29)	0 (0)	0 (0)	2 (12)	1 (8)	1 (25)
Foot heelcord contractures	3 (18)^b^	2 (14)	1 (100)	0 (0)	3 (18)	3 (23)	0 (0)
Equinovarus foot	0 (0)	0 (0)	0 (0)	0 (0)	1 (6)	1 (8)	0 (0)
Supinated foot	0 (0)	0 (0)	0 (0)	0 (0)	1 (6)	1 (8)	0 (0)
Clino-/syn-/campto-dactyly	6 (35)	6 (43)	0 (0)	0 (0)	4 (24)	3 (23)	1 (25)
*Others*							
Small joint laxity	7 (41)	5 (36)	1 (100)	1 (50)	5 (29)	4 (31)	1 (25)
Congenital hip Dislocation	2 (12)	2 (14)	0 (0)	0 (0)	1 (6)	1 (8)	0 (0)
Talipes	1 (6)	1 (7)	0 (0)	0 (0)	1 (6)	1 (8)	0 (0)
Generalized muscular hipotrophy	14 (82)	12 (86)	0 (0)	2 (100)	12 (71)	8 (62)	4 (100)

^a^Coxa valga subluxans diagnosed by X-rays

^b^Of note 8/17 patients already performed Achille's heel cord lengthening at time of clinical evaluation

Overall, patients with CS harboring the Gly12Ser variant showed a more severe skeletal phenotype compared to patients carrying other *HRAS* pathogenic variants (e.g., Gly12Ala and Gly13Cys).

Scoliosis in CFCS was more prevalent compared to CS. Age of onset was after 8 years in 2/6 of patients (34%) with a slow progression over the years; whereas 4/6 of patients (66%) showed an early onset-scoliosis associated with hypotonia. Three of them were treated with a corset, and they were not able to walk. Other frequent skeletal findings were pectus anomalies and pterigium colli. Ulnar deviation of fingers, elbow contractures and cubitus valgus were also present, but they were less prevalent than in CS. Hip and knees contractures and feet anomalies (mostly characterized by pes valgus and planus) were also present. In the CFCS group, patients with *MAP2K1/2* pathogenic variants showed different skeletal characteristics compared to those carrying *BRAF* variants, with a higher prevalence of scoliosis, pectus anomalies, upper limbs anomalies, hip contractures and pes valgus compared to the *BRAF* variant. Statistical analysis to compare orthopedic findings detected in CS *vs* CFCS (full cohorts) did not reach any significance for any item. At the time of clinical evaluation, coxa valga subluxans was radiologically documented in 7/8 patients with CS (88%) and 3/11 patients with CFCS and *BRAF* pathogenic variant (27%). However, one patient with CS (Gly12Ser) was radiologically negative for hip subluxation at the time of recruitment, though she was surgically treated at the age of 5 for this problem. Generalized muscular hypotrophy (small and flabby muscles both in upper al lower limbs) was detected in most of patients with CS and CFCS, with higher prevalence in CS (Table 3).

**Fig. 1:**
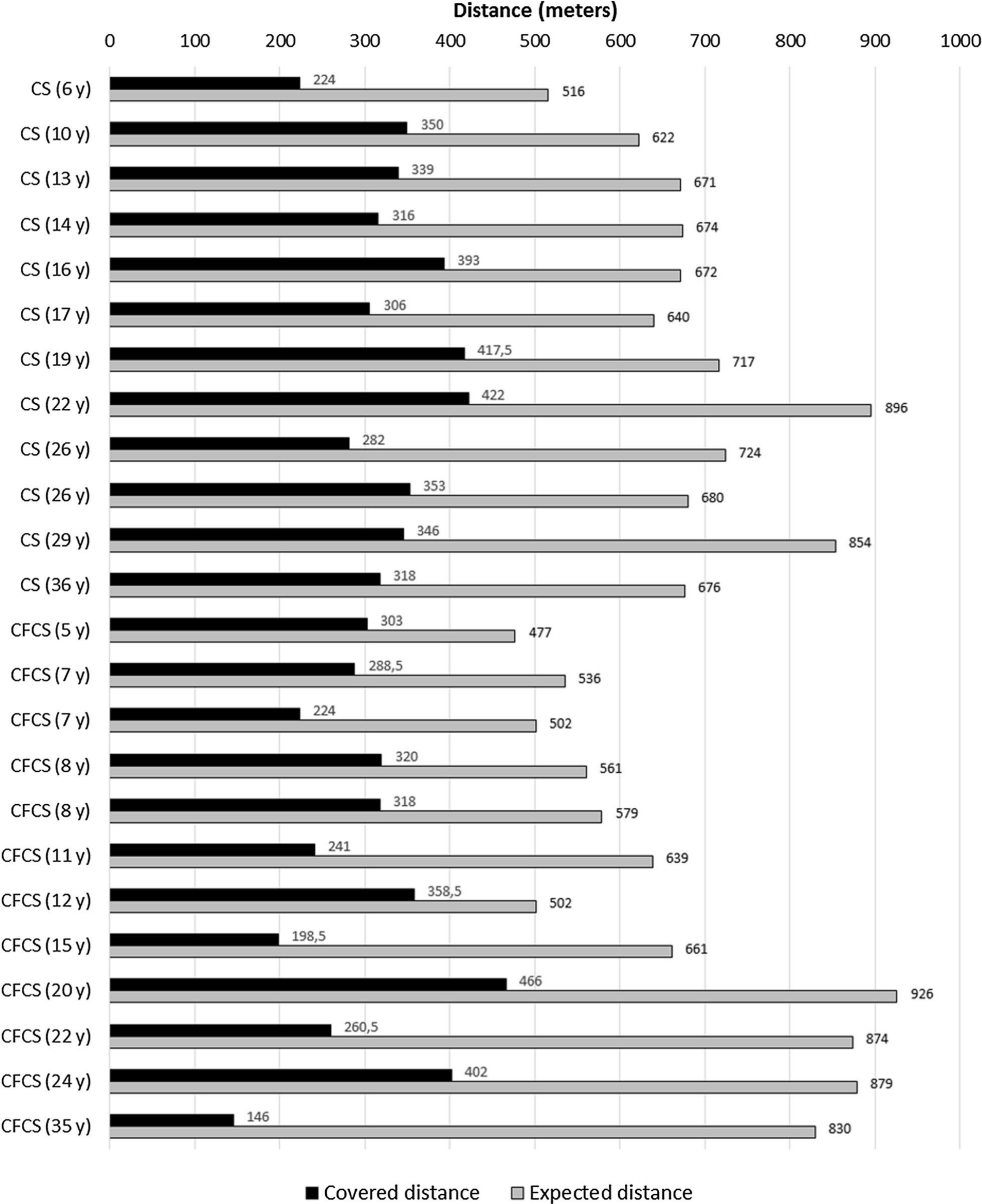
Six minute walk test in CS and CFCS

**Fig. 2:**
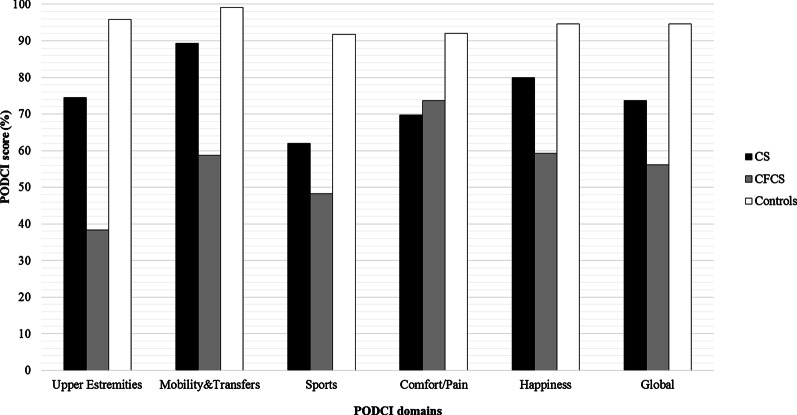
PODCI scores in Children (population under 11 years of age)

**Fig. 3:**
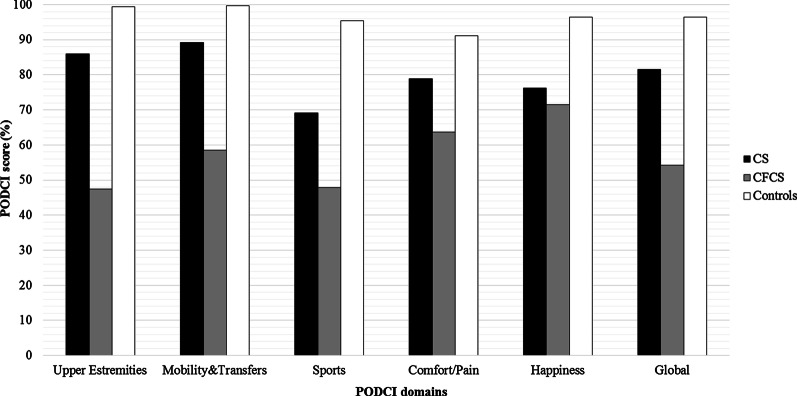
PODCI scores in Adolescents and young adults (population over 11 years of age)

Functional and disability evaluations were performed by using the 6MWT and PODCI scale, respectively. A total of 24 patients were able to perform the 6MWT (12/17 CS and 12/17 with CFCS). Among the 5 patients with CS who did not perform the test, 1/17 did not reached independent walking at the time of clinical evaluation, 2/17 had undergone surgical interventions and they were in the post-operative period, and 2/17 patients complained of hip pain due to hip dislocation. In the CFCS subcohort, the 5 patients who were not able to perform the 6MWT showed multiple musculo-skeletal deformities (particularly, a severe scoliosis was present in 4 of them) associated with loss of walking ability and severe epilepsy. Overall, the CS and CFCS groups covered on average 49% and 46% of the expected distance, respectively. No significant differences occurred between genetic analysis, phenotypes, gender or age of assessment (Fig. 1). The average PODCI scores for CFCS (both children and adolescents/young adults) were constantly lower compared to CS population (Figs. 2, 3). Statistically significant differences between CFCS and CS patients were recorded in the following domains: upper-extremities mobility (children *p* = 0.04, adolescents and young adults *p* = 0.01), transfer and basic mobility (children *p* = 0.04, adolescents and young adults *p* = 0.03) and global functioning domains (adolescents and young adults *p* = 0.02). When compared to normative values, the CFCS group (both children and adolescents/young adults’ sub-groups) reached significantly lower scores in all domains, with the exception of comfort and pain in the group of children (*p* = 0.2). Significant differences between CS and normative values were detected in all domains, with the exception of happiness both in children (*p* = 0.3) and adolescents/young adults (*p* = 0.08) (Fig. 3). The prevalence of main orthopedic findings and the comparison with the literature data are shown in Table 4.

**Table 4:** Actual main orthopedic findings and literature data review

	Present data		Reinker et al. [11]		Detweiler et al. [13]	White et al. [9]	Yassir et al. [8]	Armour et al. [10]
	CS N = 17 (%)	CFCS N = 17 (%)	CS N = 2 (%)^a^	CFCS N = 32 (%)^a^	CS N = 43 (%)	CS N = 17 (%)^b^	CS N = 18 (%)^b^	CFCS N = 38 (%)
*Spine*								
Scoliosis	5 (29)	6 (35)	1 (50)	8 (25)	25/40 (36)	10 (59)	3/18 (17)	11/33 (33)
Kyphosis	3 (18)	3 (18)	0 (0)	6 (19)	23/40 (58)	3 (18)	3/18 (17)	6/26 (23)
Lordosis	1 (6)	1 (6)	0 (0)	1 (3)	6/32 (19)	NA	NA	NA
*Upper extremities*								
Cubitus valgus	5 (29)	4 (24)	1 (50)	0 (0)	NA	NA	NA	NA
Elbow contracture	9 (53)	5 (30)	1 (50)	4 (12)	21/38 (55)	NA	10/16 (63)	NA
Ulnar deviation	14 (82)	6 (35)	NA	NA	26/41 (63)	NA	4/18 (22)	NA
*Lower extremities*								
Hip dysplasia	8/9 (89)^c^	3 (27)^c^	0 (0)	5 (16)	17/38 (45)	NA	3/18 (17)^c^	NA
Genu valgum	5 (29)	3 (18)	NA	NA	7/28 (25)	NA	NA	NA
Knee contracture	3 (18)	6 (35)	1 (50)	7 (22)	13/40 (33)	NA	NA	NA
Vertical talus	0 (0)	0 (0)	NA	NA	7/41 (17)	4 (24)	5/18 (28)	NA
Bilateral talipes equinovarus	0 (0)	3 (18)	0 (0/2)	5 (16)	1/41 (2)	1 (6)	NA	NA
Pes planus	6 (35)	6 (35)	1 (50)	20 (62)	19/36 (53)	NA	7/16 (44)	5/38 (13)
Pes cavus	6 (35)	1 (6)	0 (0)	2 (6)	3/40 (8)	4/17 (24)	NA	NA
Clino-/syn-/campto-dactyly	6 (35)	4 (24)	0 (0)	4 (12)	11/41 (27)	NA	6/16 (38)	8/38 (21)
Metatarsus varus	8 (47)	6 (35)	0 (0)	2 (6)	NA	NA	NA	NA
Hallux valgus	4 (24)	2 (12)	0 (0)	2 (6)	3/39 (8)	NA	NA	NA
*Others*								
Pectus excavatum/carinatum	9 (53)	8 (47)	0 (0)	4 (12)	12/40 (30)	8/13 (62)	1/16 (6)	19/29 (63)
Joint Laxity	7 (41)	5 (29)	NA	4 (12)	35/41 (85)	3/3 (100)	16/16 (100)	17/27 (63)
Achille tendon release	8 (47)	1 (6)	NA	NA	17/43 (40)	14/17 (82)	8/18 (44)^d^	NA

^a^In this population 1/2 patients with CS and 19/32 patients with CFCS, received a molecular confirmation

^b^Patients without genetic confirmation

^c^Coxa valga subluxans based on X-rays

^d^5 surgically treated and 3 with serial casting

## Discussion

In the present study, the musculo-skeletal phenotype and functional status of CS and CFCS were assessed considering a single unselected and relatively large monocentric cohort of patients, and genotype/phenotype correlations were explored. We have found that orthopedic manifestations are highly prevalent in both disorders. Compared with previously published observations, the different prevalence of these features could be related to the different ages of patients recruited in the cohorts [8–13]. As demonstrated by the need of orthosis and surgical intervention, the musculo-skeletal phenotype of CS and CFCS evolves with age; for this reason, the routinely use of functional tests could help physicians to plan a timely treatment before worsening of autonomy skills.

According to the medical literature, approximately 80% of mutations in CS result in a Gly12Ser missense change, which is associated with the classic CS phenotype [3]. Based on current evidence and on our analysis, we speculate that CS patients carrying the Gly12Ser variant show a higher prevalence of orthopedic abnormalities compared to subjects with other pathogenic variants in *HRAS.* The Gly13Cys mutation shows a milder orthopedic phenotype. This adds further information about this cohort of patients with CS. Moreover, this finding is in line with previous observations demonstrating a milder neurodevelopmental impairment, a better growth outcome, and a lower risk for malignant tumors compared to the classical Gly12Ser variant [3, 22]. On the same way, our study documents that patients with CFCS harboring the *MAP2K1/2* pathogenic variants generally show a more severe skeletal phenotype compared to other CFCS patients, with a higher prevalence of scoliosis and multiple joint contractures.

Limitations of this study were certainly the relatively small sample of patients with uncommon pathogenic variants and different ages of subjects recruited, even though most patients were older than 8 years. In order to confirm these data, we recommend future multicentric studies, including larger cohorts of patients with the less frequent *HRAS* and *MAP2K1/2* variants.

The prevalence of congenital bone deformities detected in our sample was low; although the high prevalence of coxa valga subluxans in our CS population suggests that orthopedic phenotype may evolve in the long-term, highlighting the importance of performing hip X-ray during infancy to rule out this possible picture. In our sample, some deformities, such as pes planus and tight Achille’s heel cord, were surgically treated, and therefore some specific items could be underestimated.

Both CFCS and CS patients frequently required use of orthosis and postural/mobility devices to improve posture and facilitate acquisition of neurodevelopmental milestones. In particular, CFCS patients reported a higher need of stroller and corset, as they had a higher incidence of scoliosis and lower limb contractures (especially knee and hip contractures), as already reported in the medical literature [11]. In our cohort, these deformities showed a progressive and worsening pattern, needing surgical intervention in some cases and compromising the independent walking.

Generalized muscular hypotrophy and hypotonia were detected in most patients. Skeletal muscle hypotonia of varying degree is a universal finding in CS and CFCS, and some authors speculated that this could be related to myopathy [23]. Tidyman et al. reported muscle biopsies in a limited number of CS and CFCS patients, describing the presence of abnormal muscle fiber size and variability as one of the potential mechanisms leading to hypotonia. Due to the small number of biopsies available, the same authors concluded that since overall muscle tissue architecture was relatively intact, experiments on animal models would be essential to better define this myopathy in vivo [23]. As it has been demonstrated the vital role of the RAS/MAPK pathway in the myogenesis regulation, mainly in myoblast differentiation and proliferation [23, 24], we hypothesize that decreased muscle fiber size and hypotonia in CS and CFCS could result from inhibition of myoblast differentiation during early muscle development, and this could explain both muscle hypotrophy found in our patients and their functional status.

As expected, CS and CFCS patients showed a worse functional performance and level of disability assessed by both 6MWT and PODCI scales, compared to normative values. All patients able to walk performed 6MWT, covering less than 50% of the expected distance. Overall, the walked distance decreased proportionally to patient’s age and, in particular, patients with CFCS showed worse performances compared to CS. This finding has been also confirmed by the PODCI scale. Global functioning items between children with CS and CFCS were not significantly different, and we speculate that this could be related to the high level of global care assistance needed by children with these genetic conditions. The lower PODCI scale scores collected in CS and CFCS compared to normative data suggest how cognitive impairment, complex clinical history, neurological condition, and musculo-skeletal deformities have a great impact on the disability in RASopathies. Data on functional and disability status published by Johnson et al. detected lower scores in CS compared to CFCS [15]*.* Interestingly, both Johnson’s and our study did not detect a significant difference in the comfort and pain domains between syndromic patients and normative values. We believe that parents’ report could represent a crucial bias, since detection of pain in patients with intellectual disability is quite challenging [25]. On the same way, the absence of significant differences between happiness domain in CS *vs* normative values could be related to the friendly behavior shown by patients with CS [26].

Overall, the musculo-skeletal manifestations in CS and CFCS should be included in a more comprehensive clinical assessment, in which growth and neurodevelopmental delay have to be considered as universal features. We suggest that worsening of the neurological profile in terms of behavioral pattern and epilepsy in CFCS [27] and the abnormal posture and bone health in CS [28, 29] may contribute to the definite outcome, justifying the lower PODCI scores found in our patients compared to normatives*.* Results of the 6MWT and PODCI scales underscore how the routinely assessment of disability status could be useful to evaluate the functional impact of orthopedic and neurological manifestations on patients’ outcome, monitor their worsening and plan a tailored treatment of comorbidities.

The adequate classification of musculo-skeletal deformities and their overtime monitoring together with disability status assessment could represent issues to assess in the frame of future clinical trials related to CS and CFCS.

## Conclusions

Previous studies focused on the prevalence of musculo-skeletal phenotype in CS and CFCS, highlighting the wide clinical variability of orthopedic malformations in these syndromes and their frequent overlap. This paper adds further information about the impact that multiple orthopedic abnormalities have on the functional status. The routinely use of both 6MWT and PODCI scale together with the orthopedic evaluation would be relevant to monitor the progression and impact of orthopedic malformations on disability along life and to plan a timely treatment (conservative or surgical). Early use of the orthosis is also suggested to reduce the progressive onset and worsening of contractures, especially in the lower limbs, and consequently reduce surgical indications. When necessary, surgical treatment should be performed as early as possible to allow minimally invasive or low impact surgical techniques. In fact, the severe progressive worsening of such deformities may lead to complex orthopedic surgery often unsustainable for these fragile patients. In this view, the precocious classification of musculo-skeletal deformities and their careful monitoring over time is of crucial relevance.

## Supplementary Information

**Additional file 1: Table S1.** Musculo-skeletal items evaluated in our sample.

## Data Availability

Data available from the corresponding author on request from physicians.

## Abbreviations

CS: Costello syndrome
CFCS: Cardio-facio-cutanoeus syndrome
NS: Noonan syndrome
6MWT: 6-Min walking test
PODCI: Pediatric Outcomes Data Collection Instrument
